# Apparent diffusion coefficients (ADC) in response assessment of transarterial radioembolization (TARE) for liver metastases of neuroendocrine tumors (NET): a feasibility study

**DOI:** 10.1177/02841851211024004

**Published:** 2021-07-05

**Authors:** Maria Katharina Ingenerf, Homeira Karim, Nicola Fink, Harun Ilhan, Jens Ricke, Karla-Maria Treitl, Christine Schmid-Tannwald

**Affiliations:** 1 Klinik und Poliklinik für Radiologie, Klinikum der Universität München, LMU München, Munich, Germany; 2 Department of Nuclear Medicine, University Hospital, LMU Munich, Munich, Germany

**Keywords:** Abdomen, gastrointestinal, magnetic resonance diffusion, perfusion, liver, treatment effects, radiation therapy, oncology

## Abstract

**Background:**

In patients with hepatic neuroendocrine tumors (NETs) locoregional therapies such as transarterial radioembolization (TARE) are increasingly applied. Response evaluation remains challenging and previous studies assessing response with diffusion-weighted imaging (DWI) have been inconclusive.

**Purpose:**

To perform a feasibility study to evaluate if response assessment with quantitative apparent diffusion coefficient (ADC) in patients with liver metastases of NETs after TARE will be possible.

**Material and Methods:**

Retrospectively, 43 patients with 120 target lesions who obtained abdominal magnetic resonance imaging (MRI) with DWI 39±28 days before and 74±46 days after TARE were included. Intralesional ADC (ADC_min_, ADC_max_, and ADC_mean_) were measured for a maximum number of three lesions per patient on baseline and post-interventional DWI. Tumor response was categorized according to RECIST 1.1 and mRECIST.

**Results:**

TARE resulted in partial remission (PR) in 23% (63%), in stable disease (SD) in 73% (23%), in progressive disease (PD) in 5% (7%) and in complete response (CR) in 0% (1%) according to RECIST 1.1 (mRECIST, respectively). ADC values increased significantly (*P*<0.005) after TARE in the PR group whereas there was no significant change in the PD group. Post-therapeutic ADC values of SD lesions increased significantly when evaluated by RECIST 1.1 but not if evaluated by mRECIST. Percentual changes of ADC_mean_ values were slightly higher for responders compared to non-responders (*P*<0.05).

**Conclusion:**

ADC values seem to represent an additional marker for treatment response evaluation after TARE in patients with secondary hepatic NET. A conclusive study seems feasible though patient-based evaluation and overall survival and progression free survival as alternate primary endpoints should be considered.

## Introduction

At the time of diagnosis, 70% of patients with neuroendocrine tumors (NETs) have metastases primarily affecting the liver (1). Surgical resection as a cure is only achievable in around 10% of patients with hepatic metastases (2). Transarterial radioembolization (TARE) is based on a high-energy beta particle emitter that is administered intra-arterially to the hepatic lesions achieving reported response rates of about 63% in the therapy of patients with secondary hepatic NETs (3,4).

Criteria for imaging response are traditionally based on changes in tumor size, most commonly according to the Response Evaluation Criteria In Solid Tumors (RECIST 1.1.); however, these criteria were initially developed to evaluate treatment response to cytotoxic therapies. Despite their broad use, growing evidence indicates that the evaluation of tumor size only is of limited value, especially when assessing the response to new treatment strategies (5,6). Morphologic changes such as necrotic or fibrotic transformation of residual viable tumor tissue represent a particular problem after locoregional therapies (LRT) and might lead to underestimation of treatment response (7,8).

Therefore, a modified version of the RECIST criteria (mRECIST) was published for hepatocellular carcinoma (HCC), taking into account the decrease of arterial hyperenhancement indicating tumor necrosis (9).

In addition, the evaluation of quantitative parameters such as apparent diffusion coefficient (ADC) values of diffusion-weighted imaging (DWI) or standard uptake value (SUV) using positron emission tomography (PET) was reported to be useful in response assessment after radioembolization for different primary tumors (10–13).

ADC values in solid tumors and hypovascular hepatic metastases were shown to increase shortly after systemic and LRTs and correlated with tumor-size changes. The extent of ADC changes was associated with overall survival (OS) and was even shown to precede anatomic changes (10–12,14–16).

To the best of our knowledge, there is a lack of larger studies that have evaluated the use of ADC quantification for monitoring treatment response of NET after TARE. The aim of the present study was to further analyze how ADC values change after TARE in hepatic metastases of NET, to evaluate if ADC changes correlate with tumor response according to RECIST 1.1 and mRECIST, and whether a conclusive study will be possible.

## Material and Methods

### Patients

In this retrospective study, 43 consecutive patients (24 men, 19 women; mean age = 64 ± 11 years) with hepatic metastases of NET of different primary tumor sites who underwent TARE at our department between August 2013 and October 2017 and had one pre-interventional and one post-interventional magnetic resonance imaging (MRI) scan on a 1.5-T scanner were analyzed. Patients with severe motion artefacts and a lesion size < 1 cm were excluded.

The study was approved by the local research ethics committee and the need for written informed patient consent was waived.

### Transarterial radioembolization

All patients included in the present study underwent TARE based on consensus in an interdisciplinary tumor conference. TARE was performed as described elsewhere (6,17). To summarize, before TARE, each patient underwent a hepatic angiography and a liver-to-lung shunt study to evaluate their suitability for TARE. Aberrant vessels were embolized with coils before injection of an average amount of 150 MBq of Technetium-99m-macroaggregated albumin into the target vessels in order to simulate the flow pattern for the therapy session. Subsequent planar and single photon emission computed tomography (SPECT) imaging was performed to exclude relevant extrahepatic sphere deposition and to evaluate pulmonary shunting. Prescribed activity was calculated according to the body surface area (BSA) method in agreement with international consensus guidelines (18). During the treatment session a microcatheter was selectively placed at the previously defined target artery and a suspension of resin spheres (SIR-Spheres®; Sirtex Medical Limited, North Sydney, NSW, Australia) in sterile water was injected. These spheres were labelled with Yttrium90, to achieve high doses of radiation at the target.

### MRI

All patients were positioned supine in a 1.5-T MR system (Magnetom Avanto, Magnetom Aera; Siemens Healthcare, Erlangen, Germany). A phased-array coil was utilized for signal reception. The routine MR protocol consisted of unenhanced T1-weighted (T1W) gradient-echo (GRE) (2D Flash) sequences in- and out-of-phase, a single-shot T2-weighted (T2W) sequence (HASTE), T1W 3D GRE sequences with fat suppression (VIBE) before and 20, 50, and 120 s (depending on circulation time) after intravenous contrast injection (Gd-EOB-DTPA; Primovist, Eovist, Bayer Schering Pharma, Germany; 25 µmol/kg body weight), a multishot T2W turbo spin echo sequence with fat saturation, diffusion-weighted sequences with b-values of 50, 400, and 800 s/mm^2^ and, after a delay of 15 min, an additional T1W GRE sequence with fat saturation (2D FLASH) and a fat-suppressed T1W VIBE 3D GRE sequence identical to those performed earlier. Parallel imaging with an acceleration factor of 2 was utilized for all sequences. ADC maps were computed from acquired DWI-MR images including all b-values. Detailed sequence parameters are provided in Table 1.

**Table 1.** Sequence parameters (Magnetom Aera and Magnetom Avanto).

Sequence and parameters	T2W SSFSE	DW-MRI	T1W 3D GRE FS pre- and dynamic post-contrast
Parallel imaging	2	2	2
Fat saturation	No	Yes	Yes
Respiratory state	Free-breathing	Respiratory gated	Inspiration
TR (ms)	800	2800 **(2300)**	3.35
TE (ms)	84 **(54)**	66 **(70)**	1.19
TI (ms)	–	–	–
FA (°)	180	180	15
FOV	380 mm, 100% **(380 mm, 75%)**	400 mm, 75% **(400 mm, 65%)**	360 mm, 75% **(400 mm, 75%)**
Matrix	320 × 320 **(320 × 189)**	192 × 130 **(192 × 113)**	256 × 154
Slice orientation	Transverse	Transverse	Transverse
Slice thickness (mm)	6	6	3
Slice gap (mm)	0.6	0.6	No gap
No. of slices	35	30	64 **(56)**
Bandwidth (Hz/pixel)	710 **(446)**	1370	450
k-space sampling	Linear	All k-space lines are measured in one TR	Line by line, time to center 6.5 s
Acquisition time (s)	*	*	21 **(19)**
b-value (s/mm^2^)	–	50, 800	–

Parameters of 1.5-T Magnetom Avanto deviating from Magnetom Aera in bold and brackets.

* Acquisition time depends on the individual patient’s respiratory rate.

FA, flip angle; FOV, field of view; GRE FS, gradient-echo fat-saturated; SSFSE, single shot fast spin echo.

### Image analysis

#### Target lesion selection

All pre-interventional MRI data were reviewed by two radiologists in consensus (Observers 1 and 2 with 2 and 12 years of experience in abdominal MRI, respectively). They defined a maximum number of three lesions per patient (each lesion > 1 cm), which were treated by TARE, and recorded the location of the lesions where they appeared best measurable.

#### ADC and size measurements

The review was conducted in two separate sessions by two readers independently: (i) pre-interventional MRI; and (ii) post-interventional MRI with a two-week interval between the review sessions.

For ADC measurements of the target lesions, circular regions of interest (ROIs) were manually drawn on the slice with the largest tumor extent on DW images while excluding neighboring structures or regions close to the rim of the lesion to avoid partial volume effects. Then, ROIs were transferred to the same slice of the ADC map to calculate intralesional ADC values including minimal (ADC_min_), maximal (ADC_max_), and mean (ADC_mean_) ADC values (below noted as 10^−3^ mm^2^/s).

ADC measurements were repeated for the same lesions on follow-up MRI images. In addition, mean ADC values of tumor-free hepatic parenchyma were measured on pre- and post-interventional DWI-MR images by placing circular ROIs, as large as possible, in areas of normal liver parenchyma. Size measurements were performed on T1W postcontrast imaging at liver specific phase on the slice with the largest tumor extent. Only in three patients were size measurements performed on T1 pre-contrast images since administration of contrast medium was contraindicated.

### Standard of reference and response to treatment

The diagnosis of NETs was established by histopathology and for most patients Ki-67 labeling index and tumor grade according to WHO were obtained. Clinical, interventional, and surgical records were collected by both radiologists.

The evaluation of treatment response was lesion-based and in line with RECIST criteria 1.1 and mRECIST as depicted in Table 2 (9,19). Both, RECIST 1.1 and mRECIST, were used, on the one hand, to apply the currently most frequently used response criteria and, on the other, to assess the changes in hypervascularization during therapy as assessed in the literature (20).

**Table 2.** Evaluation of target lesions according to RECIST 1.1 and mRECIST.

	RECIST 1.1	mRECIST
CR	Disappearance of lesion	Disappearance of any intratumoral arterial enhancement in lesion
PR	≥ 30% reduction of LD of the lesion	≥ 30% reduction of LD of viable lesion
SD	Neither PR nor PD	Neither PR nor PD
PD	An increase of LD of ≥ 20%	An increase ≥ 20% of LD of viable lesion

CR, complete response; PD, progressive disease; PR, partial remission; SD, stable disease; LD, longest diameter.

### Statistical analysis

For statistical analysis, including baseline patient characteristics and change of ADC over time, commercially available statistical software (Prism Version 6; GraphPad, San Diego, CA, USA) was utilized for all analyses.

ADC values and size measurements by both readers were averaged for further statistical analysis. The level of statistical significance was set at *P* ≤ 0.05. Normal distribution of continuous variables was assessed by visual inspection of the frequency distribution (histogram). ADC values of normal liver parenchyma and target lesions before and after therapy were compared using a two-tailed, paired *t* test, and ADC values of target lesions between different response groups were compared using a two-tailed, unpaired *t* test, respectively. In case of a non-normal distribution, the Wilcoxon rank sum test was used. Spearman’s rank correlation analysis was used to assess inter-observer agreement of measured pre- and post-treatment ADC values. For the power calculation, we used a two-sample test (PASS Version 13.0.17; NCSS, Kaysville, UT, USA).

## Results

### Patient cohort and TARE

A total of 120 target liver lesions (mean = 2.8 target lesions per patient) in 43 consecutive patients were selected by consensus review on pre- and post-interventional MRI images. The most common primary tumor sites were gastrointestinal tract (n = 22), pancreas (n = 12), lungs (n = 6), and the liver (n = 1). Two NETs were defined as cancers of unknown origin (CUP). Regarding histology, the majority of target lesions were G2 tumors (intermediate grade) with 86 lesions, followed by low grade (G1) with 19 lesions and a few high-grade lesions (G3, n = 6). There was no histology for nine lesions. Both liver lobes were treated with TARE in 39 patients, the right lobe only was treated in four patients. The treatment of both liver lobes was performed as sequential lobar radioembolization (n = 37) or in one single session (n = 2). Baseline MRI including DWI was performed 39 ± 28 days (median = 35 days) before TARE and follow-up MRI was performed 74 ± 46 days (median = 59 days) after treatment.

### Response assessment

**Fig. 1.**
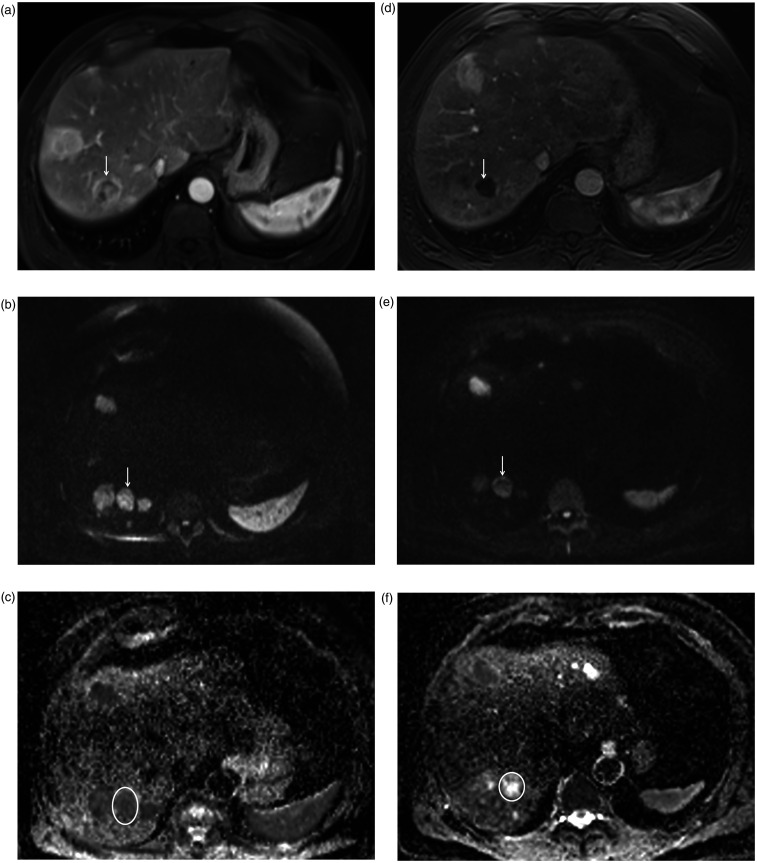
A 59-year-old man with liver metastasis of ileal NET classified as PR. (a) The pre-interventional axial contrast-enhanced T1-weighted image (arterial phase) shows a hypervascular lesion (arrow) with a diameter of 26 mm in segment 7. (b, c) The metastasis shows (b) restricted diffusion (arrow) with high signal on axial DW-MR image b = 800 s/mm^2^ and (c) dark signal (circle) on ADC map. The pre-interventional ADC values of the metastasis were 0.65 and 0.67 × 10^−3^ mm^2^/s, measured by readers 1 and 2, respectively. (d) After TARE, the metastasis (arrow) exhibited a decrease in size to 20 mm (= PR) and shows less arterial enhancement. (e) On the axial DW-MR image b = 800 s/mm^2^, the metastasis (arrow) demonstrated hyperintense signal to liver and (f) predominantly hyperintense signal (circle) on the ADC map indicating less restricted diffusion compared to the pre-interventional image. The post-interventional ADC values of the metastasis were 0.92 and 0.99 × 10^−3^ mm^2^/s, measured by readers 1 and 2, respectively. ADC, apparent diffusion coefficient; DW-MR, diffusion-weighted magnetic resonance; NET, neuroendocrine tumor; PR, partial remission; TARE, transarterial radioembolization.

**Fig. 2.**
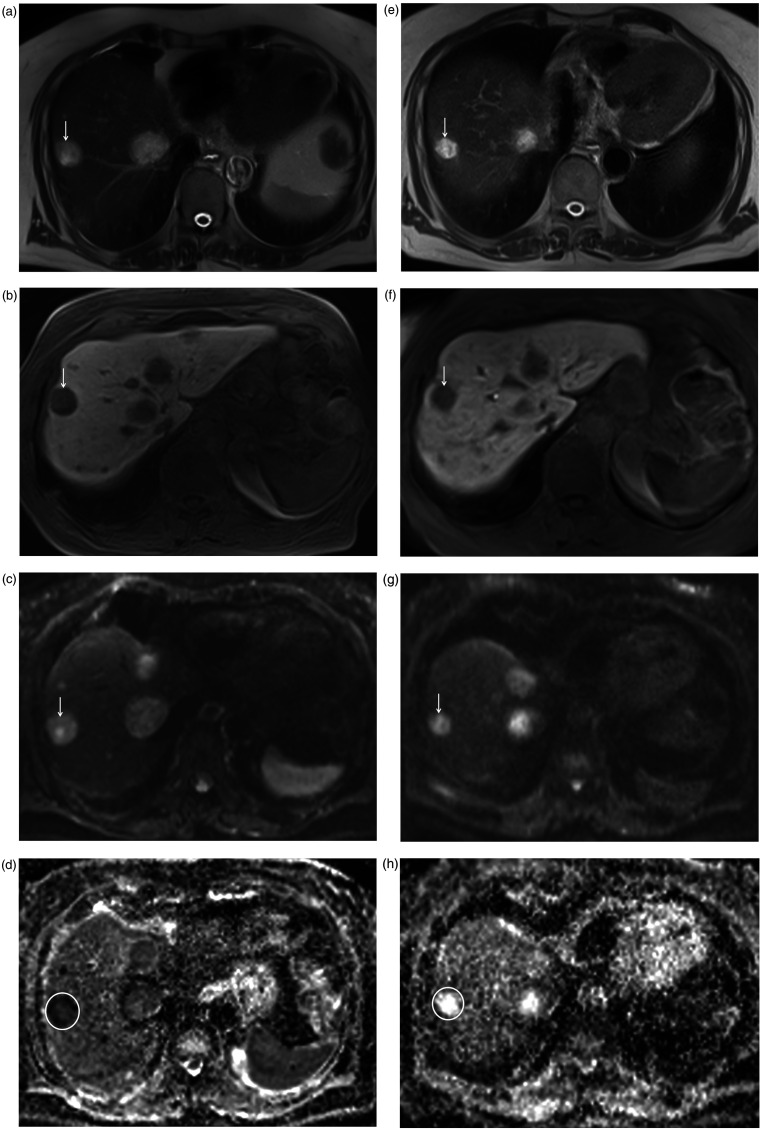
A 72-year-old women with liver metastases of ileal NET classified as SD/PR. (a) The pre-interventional axial T2W image shows an intermediate to hyperintense metastasis (arrow) in segment 8 with a LD of 21 mm on (b) contrast-enhanced T1W image (liver-specific phase). (c) The metastasis (arrow) shows restricted diffusion with high signal on axial DW-MR image b = 800 s/mm^2^ and (d) dark signal (circle) on ADC map. The pre-interventional ADC values of the metastasis were 0.60 and 0.57 × 10^–3^ mm^2^/s, measured by readers 1 and 2, respectively. (e) After TARE, the metastasis (arrow) increased in the signal on the T2W image indicating increasing necrosis, less vascularization, and therefore response to treatment. (g) Accordingly, the metastasis (arrow) showed a loss of signal on DW-MR image b=800 s/mm^2^ compared to the pre-interventional image, and (h) predominantly hyperintense signal (circle) on the ADC map indicating loss of restricted diffusion and good response to therapy. The post-interventional ADC values were 1.52 and 1.68 × 10^–3^ mm^2^/s and significantly higher compared to pre-interventional ADC values. (f) However, the metastasis (arrow) measured unchanged 22 mm cm on the axial contrast-enhanced T1W image (liver-specific phase) and was therefore rated as stable according to RECIST 1.1 criteria, while classification according to mRECIST resulted in PR. ADC, apparent diffusion coefficient; DW-MR, diffusion-weighted magnetic resonance; NET, neuroendocrine tumor; PR, partial remission; SD, stable disease; T1W, T1-weighted; T2W, T2-weighted; TARE, transarterial radioembolization; LD, longest diameter.

**Fig. 3.**
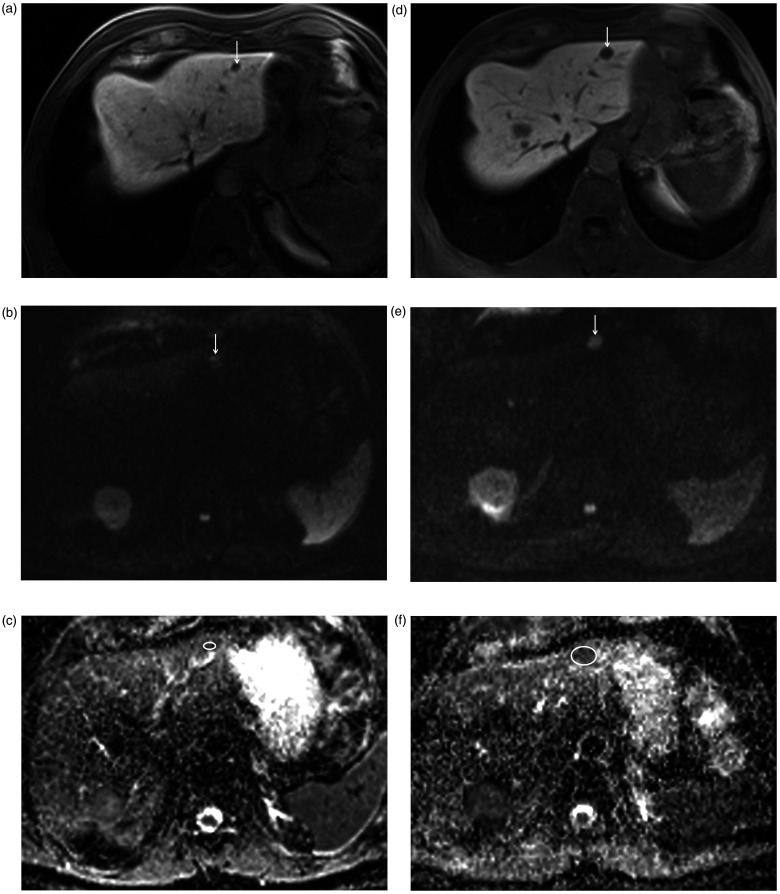
An 80-year-old man with hepatic metastasis of NET of the small bowel classified as PD. (a, d) After TARE, the metastasis (arrow) increased in size from (a) 11 mm to (d) 18 mm on the axial contrast-enhanced T1W image (liver-specific phase). (b,e) Accordingly, on both, (b) pre- and (e) post-interventional MRI, the metastasis (arrow) showed restricted diffusion with high signal on DW image with high b-value. (c) ADC values of the metastasis (circle) on pretherapeutic ADC map were 1.08 and 1.04 × 10^3^ mm^2/s^, similar to (f) the post-therapeutic scan (0.9 and 1.07 × 10^3^ mm^2^/s). ADC, apparent diffusion coefficient; MRI, magnetic resonance imaging; NET, neuroendocrine tumor; T1W, T1-weighted; TARE, transarterial radioembolization.

The mean longest diameter (LD) of all target lesions was 3.04 ± 1.59 cm on pre-interventional MR images and 2.69 ± 1.54 cm on post-interventional images. On average, the decrease of LD after treatment was 11.5% ± 18.9% (0.38 ± 0.59 cm) compared to baseline examination (*P* < 0.0001). The distribution of response assessment according to RECIST 1.1 and mRECIST is shown in Table 3. In terms of overall intrahepatic response, 13 patients showed partial remission (PR), 24 stable disease (SD), and six progressive disease (PD). In terms of extrahepatic overall response, seven patients showed PR, 26 SD, and nine PD (Figures 1-3).

**Table 3.** Distribution of treatment response according to RECIST v1.1 and mRECIST.

	RECIST 1.1	mRECIST
CR	0	1
PR	27	76
SD	87	28
PD	6	8
Not analyzed	0	7*

* Injection of contrast medium not possible.

CR, complete response; PD, progressive disease; PR, partial remission; SD, stable disease.

### ADC measurements

The mean ADC_mean_ of non-tumorous liver parenchyma for all patients was 0.96 ± 0.24 × 10^−3^ mm^2^/s on pre-interventional images and 0.99 ± 0.22 × 10^−3^ mm^2^/s on post-interventional images. There was no statistically significant change in mean ADC values of non-tumorous liver parenchyma between baseline and follow-up MRI (*P* = 0.169).

**Fig. 4.**
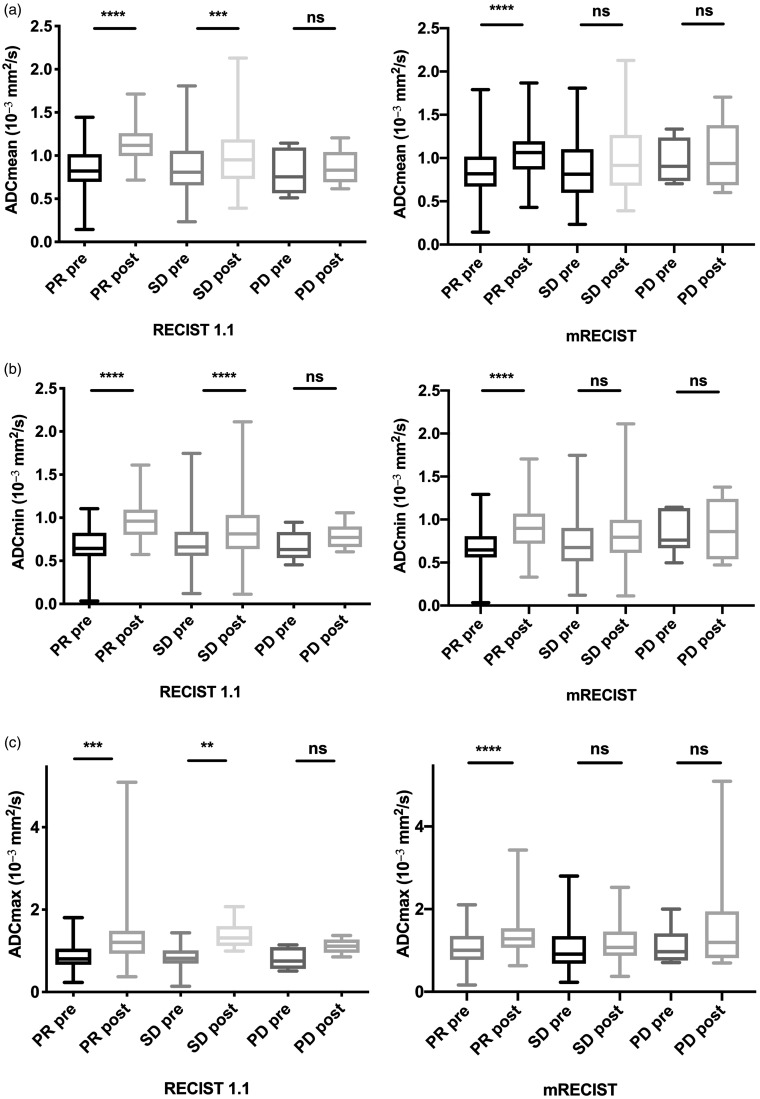
ADC values at baseline and follow-up MRI according to response groups. Box plots (median, upper and lower quartiles, maximum and minimum) displaying ADC values ((a) ADC_mean_, (b) ADC_min_, (c) ADC_max_) of target lesions before and after TARE are depicted. Lesions are divided into three response groups according to RECIST 1.1 (left) and mRECIST (right) (PD, SD, and PR). One lesion classified as CR by mRECIST was not shown for the sake of overview. ADC, apparent diffusion coefficient; CR, complete response; MRI, magnetic resonance imaging; PD, progressive disease; PR, partial remission; SD, stable disease; TARE, transarterial radioembolization.

By contrast, the mean ADC_mean_ of all target lesions increased significantly from 0.86 ± 0.31 × 10^−3^ mm^2^/s before treatment to 1.07 ± 0.52 × 10^−3^ mm^2^/s after treatment (*P* < 0.001). Regarding the three response groups according to RECIST 1.1 (PD, SD, and PR), ADC values increased significantly between baseline and follow-up examination in the PR and SD groups for ADC_min_ (*P* < 0.001 both groups), ADC_mean_ (*P* < 0.001 both groups) and ADC_max_ (*P* < 0.001 and *P* < 0.005, respectively), whereas there was no significant change of ADC values (ADC_min_, ADC_mean_, and ADC_max_) in the PD group (*P* > 0.1). According to mRECIST, ADC values increased significantly after TARE in the group of PR for ADC_min_, ADC_mean_, and ADC_max_ (*P* < 0.0001) while there was no significant change after radioembolization of ADC values in the SD (*P* > 0.2) and PD groups (*P* > 0.3) (Fig. 4). One lesion was categorized as complete response (CR) and showed an increase of ADC values (ADC_min_, ADC_mean_, and ADC_max_), which was not analyzed statistically due to the limited number in this response group. ADC values are listed in Table 4.

**Table 4.** Results of ADC measurements of metastases and liver.

		Metastases						Liver	
		ADC_mean_*	ADC_mean_^†^	ADC_min_*	ADC_min_^†^	ADC_max_*	ADC_max_^†^	ADC_mean_*	ADC_mean_^†^
SD	Pre-TARE	0.86 ± 0.32	0.89 ± 0.4	0.70 ± 0.27	0.74 ± 0.36	1.05 ± 0.49	1.07 ± 0.58	0.95 ± 0.23	0.98 ± 0.22
	Post-TARE	1.00 ± 0.34	0.99 ±0.39	0.85 ± 0.31	0.83 ± 0.38	1.3 ± 0.65	1.18 ± 0.51	0.99 ± 0.25	1.00 ± 0.28
	*P*	**0.001**	0.16	**<0.001**	0.15	**0.004**	0.25	**0.04**	0.57
PR	Pre-TARE	0.84 ± 0.27	0.85 ± 0.28	0.67 ± 0.22	0.68 ± 0.21	1.09 ± 0.39	1.06 ± 0.39	1.01 ± 0.22	0.98 ± 0.22
	Post-TARE	1.14 ± 0.23	1.07 ± 0.29	0.96 ± 0.24	0.91 ± 0.26	1.39 ± 0.3	1.36 ± 0.45	0.97 ± 0.25	1.01 ± 0.19
	*P*	**<0.001**	**<0.001**	**<0.001**	**<0.001**	**<0.001**	**<0.001**	0.19	0.14
PD	Pre-TARE	0.80 ± 0.27	0.97 ± 0.25	0.66 ± 0.17	0.85 ± 0.25	1.00 ± 0.28	1.12 ± 0.45	0.91 ± 0.29	1.04 ± 0.16
	Post-TARE	0.86 ± 0.22	1.04 ± 0.39	0.78 ± 0.16	0.88 ± 0.35	1.11 ± 0.19	1.71 ± 1.44	0.93 ± 0.20	0.94 ± 0.17
	*P*	0.296	0.25	0.097	0.62	0,154	0.32	0.65	**<0.001**
CR	Pre-TARE	–	0.73	–	0.58	–	0.89	–	1.02
	Post-TARE	–	0.97	–	0.89	–	1.09	–	0.79
All lesions (n = 120)	Pre-TARE	0.86 ± 0.31	0.86 ± 0.31	0.69 ± 0.25	0.69 ± 0.25	1.05 ± 0.46	1.05 ± 0.46	0.96 ± 0.24	0.96 ± 0.24
	Post-TARE	1.07 ± 0.51	1.07 ± 0.51	0.87 ± 0.29	0.87 ± 0.29	1.31 ± 0.58	1.31 ± 0.58	0.99 ± 0.22	0.99 ± 0.22
	*P*	**<0.001**	**<0.001**	**<0.001**	**<0.001**	**<0.001**	**<0.001**	0.169	0.169

ADC values are noted as 10^−3^ mm^2^/s.

* Treatment response according to RECIST 1.1.

† Treatment response according to mRECIST.

ADC, apparent diffusion coefficient; CR, complete response; PD, progressive disease; PR, partial remission; SD, stable disease; TARE, transarterial radioembolization.

**Fig. 5.**
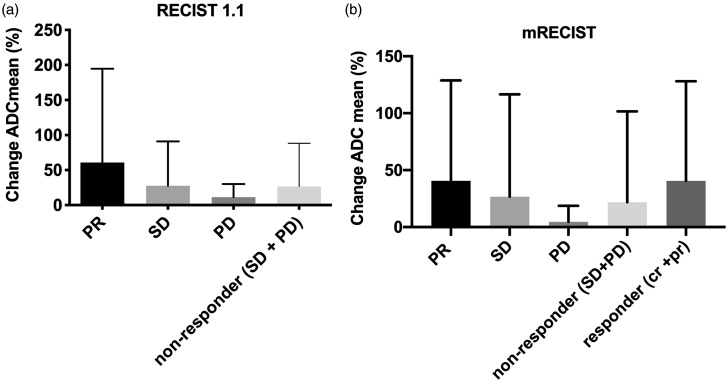
Percentage change of ADC_mean_ after TARE. Bar graphs depicting the percentage of changes (mean with SD) for ADC_mean_ values in hepatic lesions from pretherapeutic to follow-up examination with response assessment according to RECIST 1.1. and mRECIST, respectively. ADC, apparent diffusion coefficient; SD, stable disease; TARE, transarterial radioembolization.

Using mRECIST (RECIST 1.1, respectively) the average increase in ADC_mean_ values after TARE was 33% in the CR group, 41% (61%) in the PR group, 27% (28%) in the SD group, and 5% (11%) in the PD group. Applying both response classification systems, ADC_mean_ changes were slightly significantly different between responders (PR/PR and CR) and non-responders (SD and PD) (*P* < 0.05), while no significant differences were assessed between SD and PD (*P* > 0.99) (Fig. 5).

Concerning the ki-67 grading, we found no significant differences between G1, G2, and G3 lesions in tumor size, distribution among response categories, and intralesional ADC values.

The Spearman rank correlation was 0.85 as a measure for inter-observer agreement of pre-treatment ADC_mean_ values and 0.82 for post-treatment ADC_mean_ values.

## Discussion

The aim of the present study was to investigate if ADC measurements may be an additional marker assessing treatment response of hepatic metastases of NET undergoing TARE with 90Y and if a conclusive study to determine whether quantitative ADC to assess treatment response in these patients will be possible.

Our results showed that according to RECIST 1.1 and mRECIST, there was a significant increase of ADC values (ADC_min_, ADC_mean_, and ADC_max_) between baseline and follow-up examination in the PR group but not for the PD group. Second, responding hepatic lesions presented with higher ADC changes, especially of ADC_mean_, than non-responding hepatic lesions. Therefore, DWI-MR could provide important information in addition to established clinical parameters and morphological criteria to assess treatment response after TARE.

MRECIST reflects the change of vascularity of hypervascular tumors after therapy and may be useful for assessment of tumor response. Our results confirm that NET metastases show a decrease in arterial enhancement after treatment without much of a size change: According to mRECIST, many target lesions that were categorized as SD based on size only showed decreased arterial enhancement after TARE, so that the response classification was changed from SD (73% by RECIST v1.1. vs. 23% by mRECIST) to PR (23% by RECIST v1.1. vs. 63% by mRECIST). Similar results were shown by Braat et al. (21). In their study, radioembolization resulted in CR in 2%, PR in 14%, SD in 75%, and PD 9% according to RECIST 1.1 and in CR in 8%, PR in 35%, SD in 48%, and PD in 9% according to mRECIST. However, in clinical practice, the most relevant parameter remains progression to discuss a change of treatment strategy.

The change in the SD group is also reflected in the evaluation of the ADC measurements. ADC changes increased significantly in the SD group according to RECIST 1.1 whereas there was no significant increase in the SD group according to mRECIST. This might be explained by the characteristics of ADC, which inherently contain the perfusion fraction and therefore could be affected by tumor vascularity (22). Furthermore, ADC values are inversely correlated to tissue cellularity and integrity of cell membranes. After therapy, the breakdown of cell membranes and necrotic changes lead to a decrease in interstitial pressure and therefore an increase in ADC values (7,23,24).

The present study has some limitations. First is its retrospective nature. Consequently, time points of pre- and post-interventional MRI were not entirely homogeneous, which might have affected ADC measurements. However, this should not have changed general findings as the main results of this study were underlined by a good significance level. Second, we only evaluated the first follow-up MRI after TARE. Therefore, prospective trials with long-term follow-up data are necessary to confirm the findings obtained in this work and possibly integrate DWI-based therapy response evaluation in clinical routine. Prognostic evidence for DWI-based therapy response evaluation after TARE in liver metastases of colorectal and breast cancer was shown in several studies (10,11,25).

A statistical power analysis for sample size estimation based on the data obtained in our study showed that 4–5 times the number of lesions already obtained in the present study (with an alpha = 0.05 and power = 0.80) is needed for the comparison of ADC_mean_ changes in responding versus non-responding lesions. However, it should be considered when choosing for a following diagnostic test that in oncologic trials, OS is considered the gold standard and described by the European Medicines Agency (EMA) as the “most persuasive outcome,” which should represent the main objective of any antitumor treatment. However, studying a tumor entity with rare incidence and slow tumor growth such as NET, we encounter several difficulties when using OS as a primary endpoint for studies. Long survival times and the use of different post-progression treatments will influence OS as a primary endpoint. One alternative endpoint is progression-free survival (PFS), which was recommended in advanced NETs at the National Cancer Institute Neuroendocrine Tumor Clinical Trials Planning Meeting in 2011 (26). In the literature, it appears controversial if there is a significant association between PFS and OS (27) depending on the treatment.

The design of the present study serves as a feasibility study to evaluate if ADC changes correlate with tumor response according to RECIST 1.1 and mRECIST. Since our results seem to be promising, a diagnostic test with a larger patient cohort including receiver operating characteristic analysis and correlation with OS/PFS and comparison with PET/CT should be elaborated.

In conclusion, the present study shows that quantitative ADC, especially changes in ADC_mean_ values, seems to be an additional marker in assessing treatment response of secondary hepatic NETs after TARE. It may be particularly helpful for patients with a contraindication for the administration of contrast media and therefore with a lack of evaluability of other parameters besides the size such as vascularization. In addition, it could provide the oncologist with a morphometric biomarker in addition to the only size-based RECIST1.1. evaluation.
